# Body piercing and tattoo: awareness of health related risks among 4,277 Italian secondary school adolescents

**DOI:** 10.1186/1471-2458-10-73

**Published:** 2010-02-17

**Authors:** Luca Cegolon, Enrico Miatto, Melania Bortolotto, Mirca Benetton, Francesco Mazzoleni, Giuseppe Mastrangelo, VAHP Working Group

**Affiliations:** 1 Department of Environmental Medicine and Public Health, University of Padua, Padua, Italy; 2 Department of Educational Sciences, University of Padua, Padua, Italy; 3 Department of Medical and Surgical Sciences, Section of Plastic Surgery, Padua University Hospital, Padua, Italy

## Abstract

**Background:**

The awareness of health risks associated with body art among secondary school pupils has never previously been studied in depth. A large sample of secondary school adolescents from the Veneto Region (North East Italy) were investigated in order to inform health education programs.

**Methods:**

6 public secondary schools from each of the 7 Provinces of the Veneto Region were selected. All students attending the 1^st^, 3^rd^, and 5^th ^school years were surveyed by an anonymous self administered questionnaire on their perception of health risks related to body art and other explanatory variables. Logistic regression analysis was employed, reporting adjusted Odds Ratios (OR) with 95% Confidence Interval (CI).

**Results:**

Among 4,277 available students (aged 14-22 years), boys were consistently: less knowledgeable of infectious diseases related to body art (OR = 0.78; CI: 0.66, 0.94), less likely to be aware of the hygienic norms to be observed in a body art parlour (OR = 0.54; 0.44, 0.65), less likely to refer to a certified body art parlour (OR = 0.56; 0.48, 0.66), less likely to refer to a professional health care provider for complications related to body art (OR = 0.71; 0.59, 0.86). Students attending the first school year (baseline) had a lesser knowledge of body art related infectious diseases, were less likely to refer to a certified body art parlour, and to know the mandatory hygienic rules to be observed when performing body modifications. Interviewees from the provinces of Rovigo and Vicenza were less likely to be conscious of the health risks associated with body modifications, and those with tattoos were less knowledgeable about the infection risk (OR = 0.60; 0.42, 0.86) and less likely to refer to a professional health care provider in case of medical complication (OR = 0.68;0.48, 0.95). Students with piercings were less likely to refer to a certified practitioner for receiving body art (OR = 0.62; 0.50, 0.77) or therapy for medical complications (OR = 0.37; 0.29, 0.46).

**Conclusions:**

Health education programs should focus on males, pupils attending lower school years, living in specific Provinces of the Region, and with a positive attitude towards piercing or tattoo.

## Background

The number of youngsters acquiring body art has reportedly been increasing in recent years [1-3] and a list of related medical complications has been documented [1,4-10].

As the prevalence of body art has increased and the risks associated with it have become more clearly defined, medical literature has begun to explore the underlying attitudes and ideas surrounding these activities. Most of this literature pertains to undergraduate/college students, generally aged over 18 years, [11-15] risk-taking behaviour categories,[3,15-20] or particular sub-groups [21-24]. The perception of health risks from piercing and tattoo have been less frequently investigated [13,25-28].

Few studies have focused solely on secondary school pupils [19,21,28,29]. Gold [26] described adolescents' attitudes and practices toward body piercing and their awareness of the associated health risks in a convenience sample of 225 participants, aged 12-21 years; however, tattooing was not included and these subjects were recruited from an urban adolescent clinic, so were not representative of the general population. Houghton [28] investigated the level of awareness of the health risks associated with tattooing in high school students, but did not consider body piercing and the study was conducted a number of years ago and on a relatively small sample [28].

In view of the above, the present study investigated, in a sizable sample of Italian secondary school adolescents of the Veneto Region (North East Italy), the perception of the health risks associated with both piercing and tattoo. This research was undertaken to help identify priorities and to inform decisions about future investment in programs designed to educate the adolescents of the Region on the health risks associated with the practices of tattoo and piercing.

## Methods

### Sampling strategy

The study base consisted of schools. In Italy secondary school normally starts at 13-14 years of age and lasts five years. However a student can be required to repeat one or more years for poor performance, and thus may not leave secondary school until he/she is over the age of 18-19 (the expected age). From the 1st to the 5th year of study, pupils are divided into sections. Education is currently obligatory until the age of 16.

In each of the seven Provinces of the Veneto Region six schools (belonging to each of the six types of Italian public secondary schools) were chosen by convenience sampling, on the basis of individual negotiations with the respective schools' head teachers. The original sample included 42 (= 6 × 7) schools, 41 of which eventually agreed to take part. In each school two sections of pupils attending the 1^st^, 3^rd^, 5^th ^school years were randomly selected. The 4,524 students attending these classes (adolescents aged 14 to 22 years) were surveyed. All of them returned the questionnaire, but after cleaning the dataset, 4,277 interviewees (95% of the initial 4,524) were suitable for the analysis.

### Questionnaire

A structured 22-item anonymous questionnaire was used which took approximately 10 minutes to complete (Additional file 1). The questionnaire comprised questions prompting for yes/no or multiple choice on: place of residence ("city" any Province capital; "town" >15,000 inhabitants; "small town" <15,000 inhabitants); Province of residence; single-parent household; number of siblings; gender and age of siblings; father's/mother's age; education level of father/mother (low = junior secondary school, corresponding in Italy to attending school until 13 years of age; medium = secondary school; high = University or postgraduate degree); satisfaction with physical appearance (yes; fairly; no); attitude towards tattoo and piercing separately (indifferent or not interested; interested or keen to try; already experienced). Unlike boys, body piercing on a girl was defined as any piercing of the body, excluding the earlobe. Those declaring to be indifferent/not interested or interested/keen to try had never experienced piercing/tattoo before; students who already had a piercing/tattoo included those who had removed it.

The survey was trialled in a convenience sample of 100 secondary school pupils of the same Region. Based on their feedback, adjustments were made to the survey instrument to clarify instructions and the wording of the questions.

Ethical approval was obtained from the schools' head-teachers and the institutional review board of the Postgraduate Training Institution for Secondary School Teachers of the Veneto Region (SSIS Veneto), the latter being an authority body of the University of Padua. Since the questionnaire was anonymous and self completed and the subject of the interview was not intrusive, parental consent for participants younger than 18 years was waived.

### Data collection

The field survey was undertaken in 2007. In each classroom, before the interview, a researcher explained the purpose and methods of the study, the time necessary to complete the questionnaire and confirmed that the responses would remain confidential. The fact that participation was voluntary was stressed. The questionnaire was returned in a sealed envelope after its completion.

### Statistical analysis

The outcome variables were as follows:

• **Infectious diseases knowledge (Outcome 1)**. A binary variable built as follows. The question: "Which of the following diseases can be transmitted by piercing or tattoo?" included the pre-classified responses: HIV, viral hepatitis, impetigo, erysipelas, and syphilis, separately for piercing and tattoo. One point was assigned every time the student answered "yes" to each of above choices. If the final score was higher than 5 (cut-off) and the student answered "yes" to the question: "Do you think there are diseases associated with the practice of tattoo or piercing?", then Outcome 1 assumed the value 1 (and 0 otherwise).

• **Knowledge of mandatory hygienic rules related to body art (Outcome 2)**. A binary variable derived from the following question: "Please identify the indicators of professionalism that a body art operator should have". If the student gave maximum priority to these points: "adoption of single-use needles; systematic sterilization of equipment; use of latex gloves" the variable was given the value of 1, otherwise 0.

• **Propensity to refer to a professionally certified body art practitioner (Outcome 3)**: a binary variable being 1 if the respondent agreed and 0 otherwise.

• **Propensity to seek medical advice in the event of complications (Outcome 4)**: a binary variable being 1 if the interviewee agreed and 0 otherwise.

All the outcomes were investigated separately using all the same explanatory variables displayed in table 1.

**Table 1:** Frequency distribution of the 4,277 secondary school pupils by explanatory variables.

VARIABLES		No. (%)
Sex	Female	2,789 (65.18)
	Male	1,488(34.82)

Age	<16 years	1,494 (34.89)
	17-18 years	1,501 (35.12)
	19+ years	1,282 (29.99)

School year	1st	1,566 (36.61)
	3rd	1,478 (34.56)
	5th	1,233 (28.83)

Residence (missing: 179)	City centre	850 (20.74)
	City outskirt	979 (23.89)
	Town	412 (10.05)
	Small town	1,857 (45.31)

Province of residence (missing: 32)	Belluno	509 (11.97)
	Verona	674 (15.90)
	Vicenza	402 (9.46)
	Padua	739 (17.42)
	Venice	554 (13.05)
	Treviso	621 (14.60)
	Rovigo	746 (17.59)

Satisfaction with physical appearance (missing 69)	Yes	1,511 (35.93)
	Fairly	2,258 (53.65
	No	439 (10.42)

Single parent household	No	3,806 (88.99)
	Yes	471 (11.01)

No. of siblings (95 missing)	0	779 (18.63)
	1	2,349 (56.17)
	2+	1,054 (25.20)

Senior sibling of same sex	No	3,963 (92.66)
	Yes	314 (7.34)

Father's age (missing: 409)	<49 years	1,824 (47.16)
	49+ years	2,044 (52.84)

Mother's age (missing: 337)	<47 years	2,058 (52.23)
	47+ years	1,882 (47.77)

Mother's education (missing: 144)	Low	1,456 (35.23)
	Medium	2,007 (48.56)
	High	670 (16.21)

Father's education (missing: 216)	Low	1,353 (33.32)
	Medium	1,917 (47.21)
	High	791 (19.48)

Attitude towards Piercing (missing: 100)	Indifferent/Not interested	2,276 (54.49)
	Interested/Keen to try	1,061(25.40)
	Done	840 (20.11)

Attitude towards Tattoo (missing: 191)	Indifferent/Not interested	1,900 (46.50)
	Interested/Keen to try	1,928 (47.19)
	Done	258 (6.31)

Number (No.) and percentage (%).

Hypotheses comparing binary outcome variables were tested using multivariable logistic regression analysis, reporting Odds Ratios (ORs) of differences between the groups compared. The explanatory variables were selected by backwards selection using p < 0.05 as criteria. ORs were weighted for gender and age using 2007 Census data, to make the results more representative of the adolescents of the Veneto Region aged 14 to 22 years. Stata 10 software (Stata Corporation, Texas, USA) was employed using the command "xi: svy: logistic ..." followed by "swaic, model back" to obtain the minimum Akaike Information Criterion (AIC).

Subjects with missing data were retained in the dataset and excluded by the statistical software when handling a particular variable. Since the variables had different numbers of missing values and different variables entered into the final models of regression analyses, the sample sizes differ for each analysis.

## Results

Table 1 shows that strata were of roughly similar size concerning age, school year, Province of residence, mother's and father's age, satisfaction with physical appearance. Most of the students were females, resided in small towns, in families with both parents, with more than two children and with a low or medium level of socio-economical status, as shown by the educational level of their respective parents. Prevalence of body piercing was 20%, whereas tattoo was 6%. 25% of the un-pierced considered piercing, 47% of the un-tattooed considered tattooing.

56% (= 470/840) of those with a piercing were underage, the equivalent for tattoo was 48% (= 125/258). 166 individuals reported having both piercing and tattoo and 87 of these (52% = 87/166) were <18 years of age (data not shown).

Table 2 shows the results of four multivariate regression models.

**Table 2:** Weighted* multivariate logistic regression (Odds Ratio, OR, and 95% confidence interval, 95% CI) models.

INDEPENDENT VARIABLES		OUTCOME VARIABLES			
		
		(1) OR (95% CI)	(2) OR (95% CI)	(3) OR (95% CI)	(4) OR (95% CI)
Gender	Female	*Reference*	*Reference*	*Reference*	*Reference*
	Male	0.78 (0.66, 0.94)	0.54 (0.44, 0.65)	0.56 (0.48, 0.66)	0.71 (0.59, 0.86)

Age	<16 years				*Reference*
	17-18 years				1.01 (0.60, 1.72)
	19+ years				0.57 (0.28, 1.16)

School year	1st	*Reference*	*Reference*	*Reference*	*Reference*
	3rd	1.89 (2.54, 3.79)	1.54 (1.27, 1.87)	1.32 (1.11, 1.57)	1.03 (0.61, 1.74)
	5th	3.10 (2.54, 3.79)	2.29 (1.84, 2.85)	1.70 (1.41, 2.04)	1.99 (0.97, 4.08)

Province of residence	Belluno	*Reference*	*Reference*	*Reference*	*Reference*
	Verona	1.03 (0.77, 1.37)	0.76 (0.55, 1.04)	1.30 (0.98, 1.72)	1.04 (0.76, 1.41)
	Vicenza	0.82 (0.59, 1.16)	0.61 (0.43, 0.87)	0.54 (0.40, 0.73)	0.90 (0.64, 1.28)
	Padua	0.90 (0.68, 1.19)	0.92 (0.67, 1.26)	1.02 (0.78, 1.33)	1.07 (0.79, 1.45)
	Venice	0.91 (0.67, 1.23)	0.76 (0.54, 1.06)	1.06 (0.79, 1.41)	1.07 (0.77, 1.47)
	Treviso	0.91 (0.68, 1.22)	1.24 (0.88, 1.74)	1.00 (0.76, 1.33)	0.83 (061, 1.12)
	Rovigo	0.63 (0.47, 0.85)	0.73 (0.54, 1.01)	0.84 (0.64, 1.10)	0.69 (0.51, 0.93)

No. of siblings	0		*Reference*	*Reference*	
	1		0.82 (0.65, 1.03)	0.87 (0.72, 1.06)	
	2+		0.63 (0.48, 0.82)	0.79 (0.63, 0.99)	

Tattoo	Indifferent/not interested	*Reference*		*Reference*	*Reference*
	Interested/keen to try	0.88 (0.73, 1.05)		1.11 (0.94, 1.32)	1.07 (0.88, 1.28)
	Done	0.60 (0.42, 0.86)		1.02 (0.72, 1.43)	0.68 (0.48, 0.95)

Piercing	Indifferent/not interested	*Reference*		*Reference*	*Reference*
	Interested/keen to try	0.91 (0.74, 1.12)		0.82 (0.67, 0.99)	0.86 (0.69, 1.07)
	Done	1.03 (0.81, 1.30)		0.62 (0.50, 0.77)	0.37 (0.29, 0.46)

(1) = Knowledge of infectious disease risk. Model fitted on 2,678 complete observations

(2) = Knowledge of hygienic norms mandatory in tattoo parlours. Model fitted on 3,093 complete observations

(3) = Propensity to refer to a certified parlour to undergo body art. Model fitted on 3,341 complete observations

(4) = Propensity to seek medical advice in the event of complications related to body art. Model fitted on 3,341 complete observations

* ORs weighted for sex and age to make the results more representative of the adolescents of the Veneto Region aged 14 to 22 years

### Outcome 1

Males (OR = 0.78; CI: 0.66, 0.94), pupils living in the Province of Rovigo (OR = 0.63; CI: 0.47, 0.85), and those who already had a tattoo (OR = 0.60; CI: 0.42, 0.86) were less likely to have an acceptable knowledge of infectious diseases related to body art. Conversely, students attending the third (OR = 1.89; CI: 2.54, 3.79) and fifth (OR = 3.10; CI: 2.54, 3.79) school years had a better knowledge. The multivariate logistic regression model was fitted on 2,678 complete observations; the corresponding Wald chi square was 153.10 (p < 0.001), the pseudo R^2 ^0.04, and the minimum AIC was 3,544.59.

54.38% (= 1,820/3,347) of the students had a reasonable knowledge of the infectious diseases related to body art (data not shown).

### Outcome 2

Males (OR = 0.54; CI: 0.44, 0.65), pupils living in the Province of Vicenza (OR = 0.61; CI: 0.43, 0.87), and having more than one sibling (OR = 0.63; CI: 0.48, 0.82) were less likely to be aware of the statutory hygienic norms to be observed in a body art parlour. By contrast, students attending the third (OR = 1.54; CI: 1.27, 1.87) and fifth (OR = 2.29; CI: 1.84, 2.85) school years were more likely to be aware. The multivariate logistic regression model was fitted on 3,093 complete observations; the corresponding Wald chi square was 158.88 (p < 0.001), the pseudo R^2 ^0.05, and the minimum AIC was 3,435.36.

72.3% (= 2,803/3,879) of the students had a sufficient knowledge of these hygienic norms (data not shown).

### Outcome 3

Males (OR = 0.56; CI: 0.48, 0.66), students living in the Provinces of Vicenza (OR = 0.54; CI: 0.40, 0.73), and students desiring (OR = 0.82; CI: 0.67, 0.99) or already with piercings (OR = 0.62; CI: 0.50, 0.77) were less likely to refer to a certified body art parlour for receiving either a tattoo or a piercing. Students attending the third (OR = 1.32; CI:1.11, 1.57) and fifth (OR = 1.70; CI: 1.41, 2.04) school years were instead more likely to prefer a certified shop. This multivariate logistic regression model was fitted on 3,341 complete observations, the corresponding Wald chi square was 131.69 (p < 0.001), the pseudo R^2 ^0.03, and the minimum AIC was 4,188.51

63.46% (= 2,714/4,277) of the interviewees considered it important to refer to a certified body art parlour (data not shown).

### Outcome 4

Males (OR = 0.71; CI: 0.59, 0.86), students living in the province of Rovigo (OR = 0.69; CI: 0.51, 0.93), and with a piercing (OR = 0.37; CI: 0.29, 0.46) or a tattoo (OR = 0.68; CI: 0.48, 0.95) were less likely to refer to a professional health care provider in the event of medical complications related to body art. This multivariate logistic regression model was fitted on 3,341 complete observations; the corresponding Wald chi square was 158.88 (p < 0.001), the pseudo R^2 ^0.05, and the minimum AIC was 3,435.36.

73.65% (= 2,997/4,069) of the respondents would refer to a health care professional in case of complications (data not shown).

## Discussion

### Interpretation of the findings in relation to other studies

In the present study, based on a school sample of 4,277 adolescents aged 14-22 years, males appeared to be consistently less conscious about the risks of infectious diseases and mandatory hygienic norms when performing body art and were less likely to refer to a certified parlour to undergo body art and to seek medical advice in the event of related medical complications. By contrast, students attending the higher school years had a better knowledge of infectious diseases correlated with body modifications, were more likely to refer to certified body art parlours, and to know the hygienic norms mandatory in such salons. Schorzman [13] compared attitudes toward body piercing and awareness of the potential for health related problems between participants aged 17 to 20 years and those aged 21 to 25 years, between men and women and between those who had body piercing and those who had not. There were no statistical differences regarding age, sex, or race/ethnicity. Likewise, in a convenience sample of 225 participants, aged 12-21 years, recruited from an urban adolescent clinic, Gold [26] found that neither race, gender or age had a significant effect on the awareness of health risks. Houghton [27] instead, found that the highest level of awareness was among the group at greatest risk, boys with tattoos, suggesting that some males may be attracted to tattoos because of their known risks. The rationale of these conflicting findings is unclear, although an insufficient statistical power in the studies carried out by Gold, [26] Schorzman,[13] and Houghton [28] could explain this in view of the small numbers involved.

In our study, differences in awareness/education on the safest ways of practicing body art to avoid unwanted complications were found across some geographical areas. Interviewees residing in the provinces of Rovigo and Vicenza (areas more rural) had a lower perception of body art risks. We would have also expected the Provinces of Padua, Verona, and Venice to be significantly more aware of the risks associated with body art, when compared with other parts of Veneto, as the latter three Provinces are served by large Universities, thus suggesting that people living in these areas are, on average, more educated. Similar geographical analyses have never previously been carried out, due to study groups being small and spatially restricted,[11-13,26,28] whereas population-based studies failed to either investigate the awareness of health risks [1,18,29] or the geographical distribution of the findings [18,29].

In a Canadian study most secondary school students with body modifications indicated that they used the services of a body art professional,[29] a result similar to Carroll [3]. Houghton [28] instead, in a sample of Australian secondary school students, found that the majority of the tattooed participants had self-administered tattoos. An overestimation of the health risks associated with body art was reported by Schorzman,[13] despite pierced participants estimating these risks at 30% with non-pierced participants estimating them at 43% (p < 0.001). Conversely, health risks as related to body piercing and tattooing were not seen as a threat by most participants, as the majority of respondents believed they had been pierced and/or tattooed in a safe, clean environment [25]. Furthermore, Huxley [27] reported that a significant proportion of pierced and tattooed participants had not considered the health risks, while those who had were often unaware of potentially serious health problems. In our study, the majority of the respondents had a reasonable knowledge of related infectious diseases and hygienic norms to be applied in body modifications, considered it important to refer to a certified body art practitioner, would refer to a health-care professional in case of complications. However, students with tattoos were found to be less aware of the risks of the blood-borne infectious diseases potentially transmittable by body art tools. Furthermore, adolescents with a positive attitude toward body piercing (already having or considering it) were less keen to refer to a certified parlour to undergo body art, and those with piercings were less likely to seek medical advice in the event of related complications. Lastly, as body art is illegal for people younger than 18, the remarkable percentage of underage (roughly 50%) among those with piercings or tattoos could imply that both forms of body modification were performed illegally (in an unauthorized environment), or that they were carried out by the adolescents themselves or by their friends.

Such findings are important for family physicians (to better serve the body art population in their medical needs, offering preventive and tertiary interventions) and school educational staff (who can take an influential proactive role by sharing information, realistic concerns and care guidelines on tattooing and body piercing). Educators should be helped in assisting adolescents to become better informed decision makers, prevent risks and (where appropriate) dissuade them from tattooing and body piercing.

### Strengths and weaknesses

A potential weakness of this study is the fact that the schools were not randomly selected. Instead of constructing a representative random sample, a stratified convenience procedure was employed to ensure representation of all the 7 Provinces of the Region and each of the 6 types of Italian public secondary schools. Moreover collaboration and engagement with the schools' head teachers depended on individual negotiations. Convenience and ad-hoc sampling strategies are the ones most commonly used in current research on body art among adolescents [11-13,26,28].

The study base (schools) presented both advantages and disadvantages. On one hand students were given time off during school hours to complete the questionnaire, whereas had the study been based on a random sample of individuals, respondents would have been required to complete the questionnaire in their own time which would probably have reduced the response rate. On the other hand, currently education in Italy is mandatory until the age of 16, and the sample collected for this study does not include adolescents who have dropped out of school. This may lead to a slight underestimation of the phenomenon, as drop-out and street youths are proportionately more likely to adopt risk-taking behaviours and thus undergo some types of body modification [30]. Moreover our results cannot be generalised to include older populations.

As the sample exhibited a preponderance of female over male individuals, in order to improve its representativeness of the target general population the regression analysis was weighted by age and gender, using 2007 Census data of the Veneto Region.

Our definition of infectious disease knowledge is somewhat arbitrary, as we awarded the respondents a score according to what we thought were the main blood-borne diseases transmittable by body art. Each underlying disease was assigned the same weight (one point). Other infections could have been included, [10] however we selected diseases which we felt were appropriate to the educational level of the respondents.

We did not have any information on the socio-economic level of the parents as this was difficult to assess and to enquire about; however we felt that the educational level of the parents was a good proxy for socio-economic status.

The strength of the relationship (pseudo R^2 ^statistic) between the various outcomes and the explanatory variables included in the relative regression models was quite scarce. Nonetheless, thanks to the large sample size, the findings were highly significant (Wald tests).

All the four regression models had similar AICs, including the model fitted for the Outcome 1 (despite having the smallest number of complete observations).

Every variable (explanatory and dependent) with missing values was transformed into a binary term being 0 if data were missing and 1 otherwise. These binary terms and the original variables of table 1 with complete information were fitted into four models of multivariable logistic regression (where the dependent variables were the outcomes 1 to 4, separately) in order to test for the missingness pattern. As no structural pattern was noted from the distribution of missing values, imputation was not felt to be necessary. Moreover, as the maximum percentage of missing information was around 10% (solely in one variable, father's age), complete-case analysis was considered a reasonable approach.

Despite these weaknesses, our study surveyed a large number of adolescents, whereas much of the available literature is based on substantially smaller samples of undergraduate students [11-13,26]. A further strength is that our study focused on secondary school adolescents who have seldom been interviewed/questioned about body modifications [19,21,28,29] and might be a better target for health education programs on body art. Lastly, the present study is the first to investigate the health risks associated with both piercing and tattoo in this age band.

## Conclusions

Based on these preliminary results, we argue that guidance and health education should be steered towards ensuring that individuals who are more likely to obtain body art, and who are more at risk of complications, are efficiently targeted by school educators and family physicians.

Future research is recommended to compare these findings over time and geographical area (other Italian/European areas).

## What is Already Known on This Topic

The number of youngsters acquiring body art has been increasing in recent years and a list of related medical complications has been documented.

Most of the studies on body modifications are limited to small samples of undergraduate/college students, or particular sub-groups.

Secondary school adolescents have seldom been investigated on body modifications, and their awareness of the health risks related to body art has never previously been studied in depth.

## What This Study Adds

This study is the first to explore the awareness of health risks related to both piercing and tattooing in a sizable sample of secondary school students (4,277 individuals).

Males, pupils attending lower school years, living in specific Provinces of the Region, and with a positive attitude towards piercing or tattoo were less aware of the health risks related to body art. Health education programs should be designed accordingly.

## Competing interests

The authors declare that they have no competing interests.

## Authors' Contributions

LC participated in the design and interpretation of the study, collected the data, performed the statistical analysis and drafted the paper. EM, MB and MB participated in the design of the study, in the conduction of the survey, the collection of data, and organization of the research team. FM participated in the design of the study and is the guarantor. GM participated in the analysis and interpretation of the study and drafted the paper.

All authors had full access to all of the data (including statistical reports and tables) in the study and can take responsibility for the integrity of the data and the accuracy of the data analysis. All authors viewed and approved the final version and.

## Pre-publication history

The pre-publication history for this paper can be accessed here:

http://www.biomedcentral.com/1471-2458/10/73/prepub

## Supplementary Material

Additional file 1 **Questionnaire**. English version of the questionnaire administered to the secondary school pupils in the field survey. Click here for file
