# Supplementary material for: Body piercing and tattoo: awareness of health related risks among 4,277 Italian secondary school adolescents
Source: BMC Public Health. 2010 Feb 17;10:73. doi: 10.1186/1471-2458-10-73 (PMC2838811; doi:10.1186/1471-2458-10-73)
Supplement: Additional file 1 — Questionnaire. English version of the questionnaire administered to the secondary school pupils in the field survey. [file 1471-2458-10-73-S1.DOC]

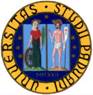
 UNIVERSITY of PADUA
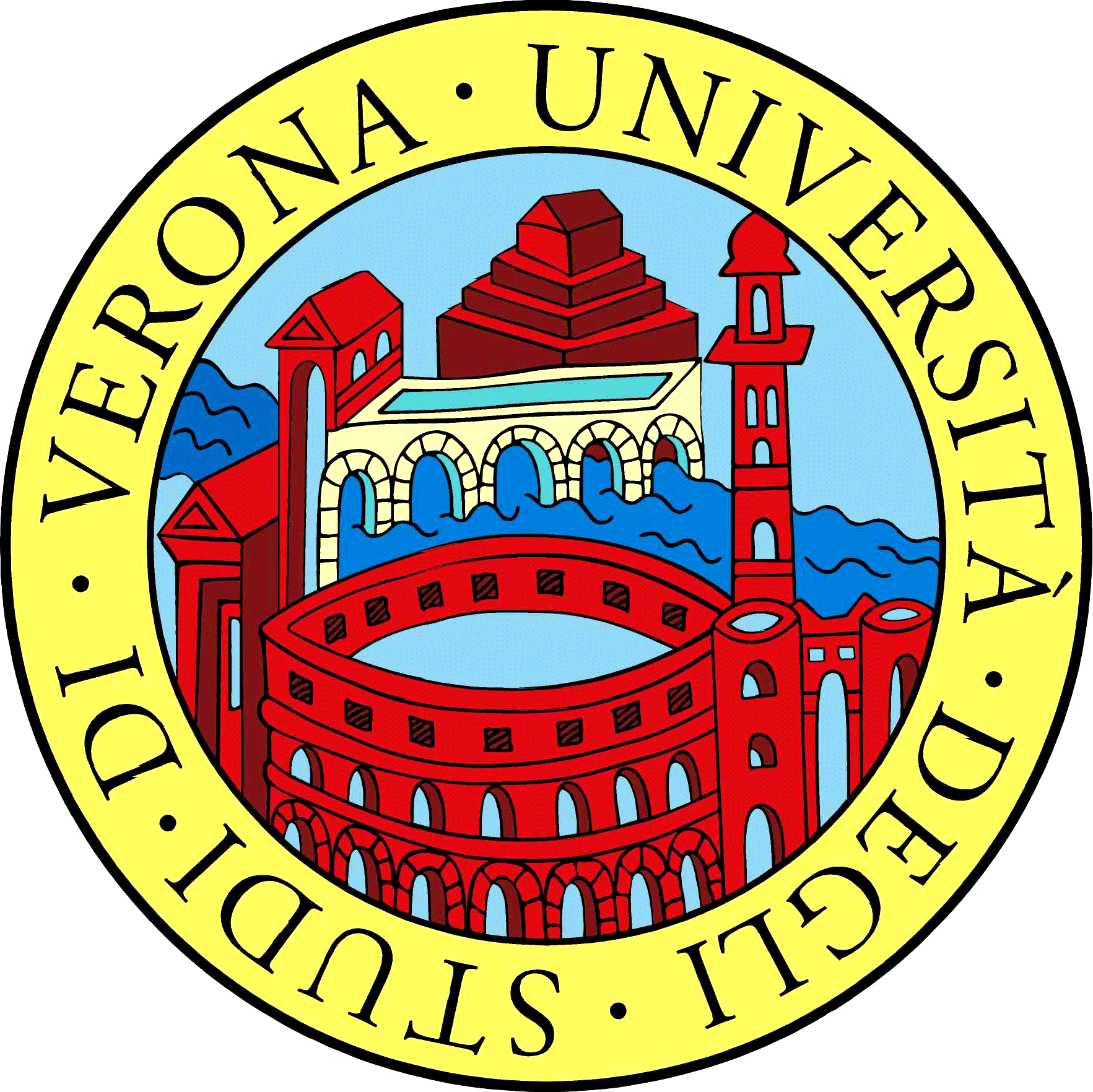


**DEPARTMENT OF ENVIRONMENTAL MEDICINE AND PUBLIC HEALTH – SECTION OF HYGIENE**

**DEPARTMENT OF MEDICAL AND SURGICAL SCIENCES – SECTION OF PLASTIC SURGERY**

**DEPARTMENT OF EDUCATIONAL SCIENCES**

UNIVERSITY of VERONA

**DEPARTMENT OF MEDICINE AND PUBLIC HEALTH**

**Socio-epidemiological survey on the practice of tattooing and body piercing among secondary school students**

**__________________________________________________________________________**

**A. GENERAL DATA**

Year of Birth **19___**

Year of study 1° ❒ 3° ❒ 5° ❒

Sex M ❒ F ❒

Nationality Italian ❒ Other _____________

Residence: City Centre ❒; City Outskirt ❒; Town (> 15000 ab.) ❒; Small Town (< 15000 ab.) ❒

Province of Residence: ____

1. **FAMILY DETAILS**

Living with

| Mother | ❒birth | ❒adoptive/step-parent |
| --- | --- | --- |
| Father | ❒birth | ❒ adoptive/ step-parent |
| Siblings | ❒birth | ❒ adoptive/ step-siblings |
| Grandparents | ❒ | |
| Uncles/aunts | ❒ | |
| Other (community…) | ❒ | |

PARENTS

|  | Born in | Nationality |
| --- | --- | --- |
| Mother | 19____ | _______________________ |
| Father | 19____ | _______________________ |

|  | Education | Employment status |
| --- | --- | --- |
| Mother | ❒ primary ❒ Secondary  ❒University/post graduate | ❒ employed ❒ self-employed ❒ unemployed ❒ retired ❒ other: |
| Father | ❒ primary ❒ Secondary  ❒University/post graduate | ❒ employed ❒ self-employed ❒ unemployed ❒ retired ❒ other: |

SIBLINGS : Have you got brothers or sisters? ❒ YES ❒ NO

|  | Year of birth | Sex | Education (highest level attended) |
| --- | --- | --- | --- |
| 1° | 19____ | M ❒ F ❒ | ❒ primary ❒ secondary ❒university |
| 2° | 19____ | M ❒ F ❒ | ❒ primary ❒ secondary ❒university |
| 3° | 19____ | M ❒ F ❒ | ❒ primary ❒ secondary ❒university |
| 4° | 19____ | M ❒ F ❒ | ❒ primary ❒ secondary ❒university |
| 5° | 19____ | M ❒ F ❒ | ❒ primary ❒ secondary ❒university |
| 6° | 19____ | M ❒ F ❒ | ❒ primary ❒ secondary ❒university |
| 7° | 19____ | M ❒ F ❒ | ❒ primary ❒ secondary ❒university |

**C. PERSONAL POSITIONS**

1. Are you satisfied with your physical appearance?

❒Yes ❒Fairly ❒No

2. What is your favourite part of your body ? ______________________________________

3. Which part of your body do you dislike? ___________________________________________

4. How much importance do you place on your physical appearance? ❒

(please give a score 1 to 10)

5. In your opinion, how much importance do other people place on physical appearance? ❒

(please give a score 1 to 10)

6. From the following list please indicate the 5 items that you value the most:

*beauty; youth; friendship; success; money; family; work; study; amusement; voluntary activities*

a.

b.

c.

d.

e.

7. Please describe yourself with an image or a metaphor:

**D. PERSONAL Information**

1. Have you undergone :

|  |  | How many *obtained* | How many *removed* | Location |
| --- | --- | --- | --- | --- |
| Piercing | ❒ No ❒Yes | _____ | _____ | ❒ Head/neck ❒ torso ❒ abdomen  ❒ arms ❒ legs   |
| Tattoos | ❒ No ❒Yes | _____ | _____ | ❒ Head/neck ❒ torso ❒ abdomen  ❒ arms ❒ legs |

1. As regards to tattooing , are you?

❒Interested ❒ Indifferent

❒Keen to try ❒Not interested

As regards to piercing, are you?

❒Interested ❒ Indifferent

❒Keen to try ❒Not interested

1. If you decided to have a piercing/tattoo this would be out of:

❒Curiosity ❒ Emulation of someone famous

❒ Peer pressure ❒A desire to improve your look

❒ Rebellion ❒Fad

❒Other _____________

1. You would like to have a piercing/tattoo but unfortunately you can’t because:

❒Your parents wouldn’t agree ❒You are afraid

❒ You can’t afford it ❒Your doctor advises you not to

❒Other _____________

1. In the event that you could not afford it, what steps would you take to find the money?

❒Saving weekly ❒Asking your family to pay

❒Working extra hours ❒Asking your friends’ for help

❒Other _____________

1. Are you aware of the difficulties involved in the removal of tattoos?

❒Yes, I know it will be difficult ❒I don’t think about them

❒I don’t think I will try to remove it ❒The problem is not going to occur

1. If you are not interested in tattooing or piercing please give the reasons why:

❒I don’t like the idea ❒I oppose this practice

❒It’s unattractive ❒It’s difficult to remove

❒Other: _____________ ❒Afraid of infection

1. **PRACTITIONER**
2. Before having a tattoo or piercing are you keen to know:

(Please rate the importance to you with a score of 1 to 6 for each question, 1 being the least and 6 being of maximum importance)

❒Who will carry out the procedure ? ❒Where will it be done? 

  ❒What are the consequences for my health? ❒ What is the cost?

❒How can it be removed? ❒ Will it be physically painful ?

1. Would you refer to:

❒Friends

❒Certified premises recommended for their standards of professionalism

❒Equipped laboratories

❒Other _____________

1. Professionalism and reliability are:

(please rate the importance to you with a score of 1 to 3 for each question, 1 being low, 2 fairly, 3 high)

❒ how good it looks ❒Needles used previously but correctly sterilised

❒Using latex gloves ❒Single-use needles only

❒Instruments sterilized and disinfected

1. **INFECTIONS / COMPLICATIONS**
2. Some diseases can be related to the practices of tattooing / piercing?

❒Yes ❒ No ❒I don’ t know

1. Why, according to a widely spread opinion, are piercing and tattooing considered less risky practices than real surgery?

❒No accurate information available

❒The needle does not penetrate as deeply

❒Operating room is not obligatory for body art

❒ No relevant consequences known

❒General anaesthesia is not mandatory

❒Do not know

1. The following diseases can be related to practices of piercing or tattooing?

| *Diseases* | *Description* | Piercing | Tattooing |
| --- | --- | --- | --- |
| HIV/AIDS | Aquired Immuno Deficiency Syndrome | ❒ Yes ❒No | ❒ Yes ❒ No |
| Viral Hepatitis | Liver infection by B or C virus (HBV / HCV) | ❒ Yes ❒No | ❒ Yes ❒ No |
| Syphilis | Infection contracted via sex or blood | ❒ Yes ❒No | ❒ Yes ❒ No |
| Impetigo | Skin infection, with or without blisters | ❒ Yes ❒No | ❒ Yes ❒ No |
| Erysipelas | Skin infection, mostly on the limbs, with strong rash and swelling | ❒ Yes ❒No | ❒ Yes ❒ No |
| Condylomata and warts | Skin prominence by Human Papilloma Virus (HPV) | ❒ Yes ❒No | ❒ Yes ❒ No |
| Glandular fever | Disease causing sore throat followed by weakness and fatigue (“kissing disease”); from Epstein Barr Virus (EBV); | ❒ Yes ❒No | ❒ Yes ❒ No |
| Herpes | Recurrent disease from Herpes Simplex Virus (HSV1); typically causing itchy blisters around the mouth. | ❒ Yes ❒No | ❒ Yes ❒ No |

1. In case of complications following piercing/tattooing what have you done?

❒Ask a friend for help ❒ Go to the GP

❒Use some disinfectant ❒Go to a hospital emergency department

❒Other _________

1. In case of complications following piercing/tattooing in the future what would you do?

❒Ask a friend for help ❒ Go to the GP

❒Use some disinfectant ❒Go to a hospital emergency department

❒Other _________
